# Patient-derived xenograft models for gastrointestinal tumors: A single-center retrospective study

**DOI:** 10.3389/fonc.2022.985154

**Published:** 2022-11-18

**Authors:** Xiongfei Yu, Yiran Chen, Jun Lu, Kuifeng He, Yanyan Chen, Yongfeng Ding, Ketao Jin, Haiyong Wang, Haibin Zhang, Haohao Wang, Lisong Teng

**Affiliations:** ^1^ Department of Surgical Oncology, The First Affiliated Hospital, College of Medicine, Zhejiang University, Hangzhou, Zhejiang, China; ^2^ Department of Medical Oncology, The First Affiliated Hospital, College of Medicine, Zhejiang University, Hangzhou, Zhejiang, China; ^3^ Department of Colorectal Surgery, Affiliated Jinhua Hospital, Zhejiang University School of Medicine, Jinhua, China

**Keywords:** patient-derived xenograft model, gastrointestinal cancer, mutational status of RAS and BRAF, drug sensitivity, clinical transformation

## Abstract

**Background:**

Patient-derived xenograft (PDX) models have shown a great efficiency in preclinical and translational applications. Gastrointestinal (GI) tumors have a strong heterogeneity, and the engraftment rate of PDX models remarkably vary. However, the clinicopathological and molecular characteristics affecting the engraftment rate still remain elusive.

**Methods:**

A total of 312 fresh tumor tissue samples from patients with GI cancer were implanted into immunodeficient mice. The median follow-up time of patients was 37 months. Patients’ characteristics were compared in terms of PDX growth and overall survival. PDX models of 3-6 generations were used for drug evaluation.

**Results:**

In total, 171 (54.8%, 171/312) PDX models were established, including 85 PDX models of colorectal cancer, 21 PDX models of esophageal cancer, and 65 PDX models of gastric cancer. Other than tumor site, histology, differentiation degree, and serum alpha-fetoprotein (AFP) level, no significant differences were found between transplantation of xenografts and patients’ characteristics. For patients who had undergone neoadjuvant therapy, the incidence of tumor formation was higher in those with progressive disease (PD) or stable disease (SD). In gastric cancer, the results showed a higher transplantation rate in deficient mismatch repair (dMMR) tumors, and Ki-67 could be an important factor affecting the engraftment rate. The gene mutation status of RAS and BRAF, two important molecular markers in colorectal cancer, showed a high degree of consistency between patients’ tumors and PDXs. However, no significant effects of these two mutations on PDX engraftment rate were observed. More importantly, in this study although KRAS mutations were detected in two clinical cases, evident tumor inhibition was still observed after cetuximab treatment in both PDX models and patients.

**Conclusion:**

A large-scale PDX model including 171 cases was successfully established for GI tumors in our center. The relationship between clinicopathological and molecular features and engraftment rates were clarified. Furthermore, this resource provides us with profound insights into tumor heterogeneity, making these models valuable for PDX-guided treatment decisions, and offering the PDX model as a great tool for personalized treatment and translation research.

## Introduction

Animal tumor model is an effective tool for preclinical efficacy and toxicity evaluation of antitumor drugs, and it can also be used to screen molecular markers related to drug efficacy prediction. Patient-derived tumor xenograft (PDX) model is an animal tumor model that is established by the engraftment of human tumors into immunodeficient mice, maintaining the characteristics of tumors. It has been proved as an effective tool for tumor biology research and for the efficacy evaluation of antitumor drugs in various tumors (1, 2). To date, several PDX models were presented for gastric cancer covering common pathological types, alpha-fetoprotein (AFP) secretion type and human epidermal growth factor receptor 2 (HER-2)-positive gastric cancer, which provided a promising tool for translation research of gastric cancer (3–5). The clinicopathological and molecular characteristics of PDX models of gastric cancer have been confirmed to be highly consistent with those of human models (6), indicating their important role in the evaluation of drug efficacy.

There were an estimated 3.5 million people who were newly diagnosed with gastrointestinal (GI) cancer, which led to the death of 2.2 million people globally in 2020 (7). Different types of GI cancer share similar endodermal developmental origins, display a spectrum of common molecular features and expose to common attacks (8). An enormous progress has been made in the last half-century in the development of non-surgical treatments. However, it is still essential to find out new biomarkers for more precisely targeting tumors. Until 2010, our team has attempted to develop PDX models for GI tumors (6, 9). Over the recent decade, based on the international modeling consensus and our team’s experience, a standardized procedure was developed for surgical sampling, specimen transfer, transplantation and tumor inoculation, cryopreservation, and resuscitation. To date, we have established nearly two hundred PDX models using tissue samples, and a number of them have been utilized for the preclinical evaluation of anticancer agents (3, 10–16).

In the present study, we analyzed clinical parameters that were associated with the engraftment rate of PDXs from patients with GI cancer to identify factors that could improve engraftment rate. We also established and assessed several PDX models to show their significance in clinical practice.

## Materials and methods

### Patients and samples

312 fresh gastrointestinal tract tumor samples from patients diagnosed with gastrointestinal tract cancer in Department of Surgical Oncology, The First Affiliated Hospital, College of Medicine, Zhejiang University were collected from January 2015 to February 2019 for the establishment of PDXs, including surgically resected specimens, endoscopic biopsy samples and needle biopsy samples. The clinical data were collected from patient records. The tumors were staged according to the eighth edition of AJCC/UICC TNM staging system. Follow-up data were obtained by phone, letter, and the out-patient clinical database (last follow-up was September 2021, median follow-up time was 37 months) and follow-up information were available in 284 patients. The overall survival (OS) time was calculated from the date of diagnosis to the last day of follow-up or the date of death. Patients derived paraffin-embedded tissue samples were used in accordance with ethical guidelines in the First Affiliated Hospital, School of Medicine, Zhejiang University (No.2018-378 and IIT20221079A). All study participants had provided informed written consent before any experiments.

### PDX establishment

**Figure 1:**
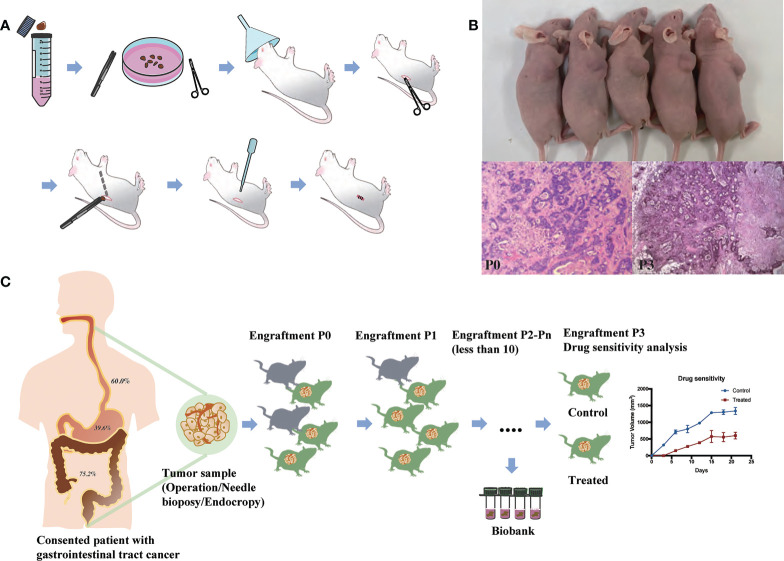
Establishment and application of patient-derived xenografts (PDXs). **(A)** Schema for the establishment of PDX models. **(B)** Representative PDX models and histology of paired patient-PDX tumors. **(C)** Engraftment rate of gastrointestinal cancers and applications of PDX models.

Four-to-six-week-old female BALB/c nude mice, purchased from Shanghai Slac Laboratory Animal Corporation (Shanghai, China), were housed with regular 12-hour light/12-hour dark cycles for at least three days before use. Ambient temperature was 20 ~ 22°C, kept at constant humidity of 40 ~ 60%. PDX models were established as in **Figure 1A**. Fresh tumor samples from patients were transported to the laboratory in complete medium (RPMI 1640 medium supplemented with 20% FBS and 0.05% penicillin/streptomycin solution) in an ice bath immediately after resection. Then tumors were transferred to a sterile Petri dish containing complete medium. Thin slices of tumor were diced into 2×2×2 mm3 pieces and washed thrice with complete medium. Under anesthesia with isofluorane, tumors were implanted into BALB/c nude mice by a small incision and subcutaneous pocket made in one side of the lower back in which one tumor piece is deposited in the pocket. While the pocket was still open, one drop of 100× penicillin/streptomycin solution was placed into the opening. We monitored xenograft growth at least twice weekly by vernier caliper measuring the length (L) and width (W) of the tumor and then removed them for serial transplantation after the volume reached about 1000 mm^3^ (**Figure 1B**). The tumor volume (V) was calculated according to the following formula: V= L×W^2^/2. Tumors were passaged no more than six times. Numerous samples from early passages were stored in the tissue bank and cryopreserved in liquid nitrogen, and used for further experiments (**Figure 1C**). Animal care and experiments were performed under the approval and supervision of the Animal Experimental Ethical Inspection of the First Affiliated Hospital, College of Medicine, Zhejiang University (No.2018-378).

### Drug sensitivity analysis

We used xenografts from the third to seventh generation for the experiments once the tumor volume reached about 150–200mm3. *In vivo* experiments were performed to evaluate the chemosensitivity, as well as the antitumor activity of several targeted drugs, including: trastuzumab, cetuximab, apatinib and bevacizumab. Experiments were ended once the tumor volume surpassed 1500mm3 or mouse weight loss reached 20%. The percentage of tumor growth inhibition (TGI) was calculated according to the following formula: TGI = (1 − T/C) × 100%, where T/C represents the relative tumor volume of treatment group and control group. After the mice had been killed, we conducted immunoblot to assess the expression of various markers.

### Mutational analysis for RAS and BRAF

All the samples from patients and PDXs were fixed with formalin and embedded in paraffin. To detect RAS/BRAF mutations, the AmoyDx KRAS/NRAS/BRAF Mutations Detection Kit (AmoyDx, Xiamen, China) approved by the China Food and Drug Administration (CFDA) was used. Based on Amplification Transformation System (ARMS) technology, the study was conducted in an accredited laboratory.

### Statistical analysis

The differences between two categorical variables were examined by Pearson’s Chi square test and Fisher’s exact tests where appropriate. Two continuous variables were compared using unpaired t-test. Non-parametric variables were compared using the Mann–Whitney test. The assumptions required to interpret the statistics have been verified using F test and QQ-plots. *P* < 0.05 was considered statistically significant. Statistical analysis was performed using SPSS 23.0 software.

## Results

### Patients’ characteristics for PDX establishment

We collected 312 fresh tumor samples from patients who were diagnosed with GI cancer (including esophageal cancer (n=35), gastric cancer (n=164), and colorectal cancer (n=113)) between 2015 and 2019, and implanted them into immunodeficient mice (BALB/c nude mice) to generate PDX models. Among 312 patients, there were 205 (65.7%) male patients, and their median age at diagnosis was 61.82 (range, 17-91) years old. Besides, 281 (90.1%) patients had primary tumors, and the remaining nine (9.9%) patients had recurrent tumors; 53 (18.9%) metastatic tumors were collected as well. Most of these specimens were obtained by surgery (289/312, 92.6%), and samples from some patients who could not undergo surgery due to the advanced tumor stage were obtained by endoscopy (9/312, 2.9%) or needle biopsy (14/312, 4.5%). There were 12 (3.8%) cases of well differentiation, 106 (34.0%) cases of moderate differentiation, and 193 (61.9%) cases of poor differentiation. Other detailed information of patients and tumor samples are summarized in **Table S1**.

### The characteristics of patients and tumors for successful growth of PDX models

PDX models were successfully generated from 171 tumor implants and were passaged for 2-6 generations. The histopathological morphology of tumor in experimental mice was consistent with the original pathological diagnosis (**Figure 1B**). The time from the implantation of fresh specimens to the first passage (maximum tumor volume, 1000 mm^3^) was 1-4 months, with an average time of 2.6 months. The overall engraftment rate was 54.8%.

**Figure 2:**
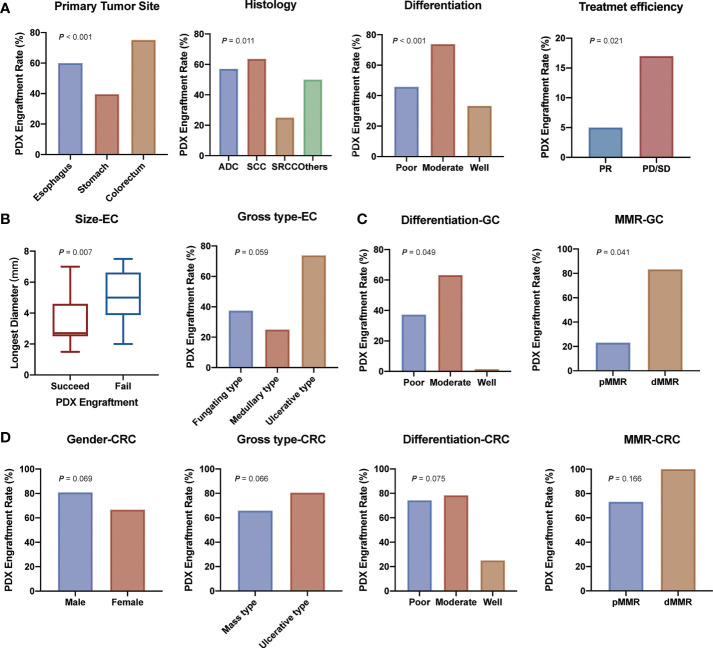
Demographic and clinical parameters associated with engraftment of gastrointestinal tract cancer patient-derived xenografts. **(A)** Clinicopathological factors related to PDX establishment. **(B)** Clinicopathological factors related to esophagus cancer PDX establishment. **(C)** Clinicopathological factors related to gastric cancer PDX establishment. **(D)** Clinicopathological factors related to colorectal cancer PDX establishment. (ADC, adenocarcinoma; SCC, squamous cell carcinoma; SRCC, signet ring cell carcinoma; PR, partial response; PD/SD, progressive disease or stable disease; EC, esophagus cancer; GC, gastric cancer; CRC, colorectal cancer).

We then analyzed clinical characteristics that affected the engraftment rates of specimens. Univariate analysis showed that several factors were significantly associated with the engraftment rate (*P* < 0.05, **Table 1** and **Figure 2**). Colorectal cancer showed a higher engraftment rate (75.2%) compared with esophageal cancer (60.0%) or gastric cancer (39.6%). Squamous cell carcinoma had a higher engraftment rate (63.6%), while signet ring cell carcinoma (SRCC) had a lower engraftment rate (25.9%) compared with adenocarcinoma (56.9%) or other histological types (50.0%). Moderately differentiated tumors had a higher engraftment rate (73.6%) compared with well-differentiated (33.3%) or poorly differentiated (46.1%) tumors. In contrast, neoadjuvant therapy was found to have no significant effect on tumor engraftment rates. However, as for patients who had received neoadjuvant therapy, the engraftment rate of progressive disease (PD) or stable disease (SD) specimens was significantly higher than that of partial response (PR) specimens (65.4% *vs*. 29.4%, *P* = 0.021, **Figure 2A**).

**Table 1:** Clinicopathological factors related to PDX establishment.

Factors	PDX succeed (n=171)	PDX fail (n=141)	*χ*2	P value
**Age**				
**Mean (year)**	62.25	61.3	–	0.528^a^
**Gender**				
**Male**	116(56.6%)	89(43.4%)	0.763	0.403^b^
**Female**	55(51.4%)	52(48.6%)		
**Primary tumor site**				
**Esophagus**	21(60.0%)	14(40.0%)	34.637	<0.001^b^
**Stomach**	65(39.6%)	99(60.4%)		
**Colorectum**	85(75.2%)	28(24.8%)		
**Sample collection method**				
**Operation**	160(55.4%)	129(44.6%)	0.650	0.755^c^
**Endoscopy**	4(44.4%)	5(55.6%)		
**Needle biopsy**	7(50.0%)	7(50.0%)		
**Sample source (Primary or metastasis)**				
**Primary tumor**	138(54.5%)	115(45.5%)	0.037	0.885^b^
**Metastasis tumor**	33(55.9%)	26(44.1%)		
**Sample source (Primary or recurrence)**				
**Primary tumor**	152(54.1%)	129(45.9%)	0.584	0.456^b^
**Recurrence tumor**	19(61.3%)	12(38.7%)		
**Treatment before biopsy**				
**No**	149(55.4%)	120(44.6%)	0.268	0.361^b^
**Yes**	22(51.2%)	21(48.8%)		
**Distant metastasis status**				
**No**	103(52.8%)	92(47.2%)	0.858	0.671^b^
**Single**	46(59.0%)	32(41.0%)		
**Multiple**	21(55.3%)	17(44.7%)		
**NA**	1	0		
**Tumor size (longest diameter)**				
**Mean (mm)**	5.29	5.43	–	0.663^a^
**Histology**				
**Adenocarcinoma**	140(56.9%)	106(43.1%)	10.627	0.011^c^
**Squamous cell carcinoma**	21(63.6%)	12(36.4%)		
**Signet ring cell carcinoma**	7(25.9%)	20(74.1%)		
**Others**	3(50.0%)	3(50.0%)		
**Differentiation**				
**Poor**	89(46.1%)	104(53.9%)	23.225	<0.001^c^
**Moderate**	78(73.6%)	28(26.4%)		
**Well**	4(33.3%)	8(66.7%)		
**NA**	0	1		
**T stage**				
**0**	1(25.0%)	3(75.0%)	7.027	0.123^c^
**1**	4(30.8%)	9(69.2%)		
**2**	19(51.4%)	18(48.6%)		
**3**	107(59.8%)	72(40.2%)		
**4**	39(50.0%)	39(50.0%)		
**NA**	1	0		
**N stage**				
**0**	60(58.8%)	42(41.2%)	4.65	0.200^b^
**1**	41(58.6%)	29(41.4%)		
**2**	36(56.3%)	28(43.8%)		
**3**	33(44.0%)	42(56.0%)		
**NA**	1	0		
**M stage**				
**0**	126(52.7%)	113(47.3%)	1.798	0.226^b^
**1**	45(61.6%)	28(38.4%)		
**TNM stage**				
**I**	11(40.7%)	16(59.3%)	4.078	0.256^b^
**II**	50(57.5%)	37(42.5%)		
**III**	64(52.0%)	59(48.0%)		
**IV**	46(61.3%)	29(38.7%)		
**Ki-67**				
**>60%+**	21(45.7%)	25(54.3%)	3.222	0.081^b^
**≤60%+**	6(24.0%)	19(76.0%)		
**NA**	144	97		
**Serum AFP level**				
**≤20ng/ml**	165(56.3%)	128(43.7%)	7.208	0.012^c^
**>20ng/ml**	1(11.1%)	8(88.9%)		
**NA**	5	5		
**Serum CEA level**				
**≤5ng/ml**	111(55.0%)	91(45.0%)	0.000	>0.999^b^
**>5ng/ml**	55(55.0%)	45(45.5%)		
**NA**	5	5		
**Serum CA199 level**				
**≤37U/ml**	132(55.0%)	34(54.8%)	0.001	>0.999^b^
**>37U/ml**	108(45.0%)	28(45.2%)		
**NA**	5	5		
**Serum CA125 level**				
**≤35U/ml**	134(53.6%)	31(60.8%)	0.883	0.360^b^
**>35U/ml**	116(46.4%)	20(39.2%)		
**NA**	5	5		

NA, not available.

^a^Unpaired two-tailed t test, ^b^Pearson’s Chi square test, ^c^Fisher’s exact test.

We further analyzed the differences between transplantation rate and clinical characteristics in esophageal cancer, gastric cancer, and colorectal cancer separately. In esophageal cancer, smaller tumors and ulcerative type tumors had a higher engraftment rate (**Figure 2B** and **Table S2**). In addition, samples from recurrent tumors tended to have a higher engraftment rate (60.0% *vs*. 37.6%) in gastric cancer (**Figure 2C** and **Table S3**). Specimens from male patients (80.9% *vs*. 66.7%), ulcerative type tumors (80.6% *vs*. 65.9%), poorly and moderately differentiated tumors (75.0% and 78.1% *vs*. 25.0%) seemed to have a higher engraftment rate (**Figure 2D** and **Table S4**) in colorectal cancer.

### Molecular parameters for successful establishment of PDX models

Subsequently, we analyzed the relationship between the engraftment rate and some tumor biomarkers, including serum tumor markers (AFP, carcinoembryonic antigen (CEA), cancer antigen 199 (CA199), and cancer antigen 125 (CA125)), immunohistochemical markers (Ki-67, HER-2, and MMR status), as well as the mutational status of RAS and BRAF genes, and molecular therapeutic targets of colon cancer. The results showed that serum tumor biomarkers were not associated with the successful establishment of PDX models except for AFP (*P* = 0.021, **Table 1**). For Ki-67, we found that a high Ki-67-positive rate (>60%+) was associated with a higher engraftment rate than a low Ki-67-positive rate (45.7% *vs*. 24.0%, *P* = 0.081, **Table 1**). Transplantation rates of samples from deficient mismatch repair (dMMR) tumors were higher than those from proficient mismatch repair (pMMR) tumors both in gastric cancer (83.3% *vs*. 23.1%, *P* =0.041, **Figure 2** and **Table S3**) and colorectal cancer (100% *vs*. 73.2%, *P* =0.166, **Figure 2** and **Table S4**). As for the important therapeutic target HER-2 in gastric cancer, the correlation between HER-2 status and PDX engraftment was not identified (**Table S3**). Besides, 46 PDX tissues of colorectal cancer were detected with the mutation status of RAS/BRAF genes, including 24 patients with RAS mutation and five patients with BRAF mutation. The correlation between mutation status and PDX engraftment rate was not found. There was also no significant association between RAS mutation and survival outcomes. The detailed mutation data of the 46 colorectal cancer-associated PDX models are presented in **Table S5**.

### Application of individualized therapy in the PDX models

One of the most important elements to evaluate the PDX models is the therapeutic response. In present study, we present two representative colon cancer patients with KRAS mutations who received individualized therapy using the PDX models. The detailed data of these two patients are shown in **Table S6**.

**Figure 3:**
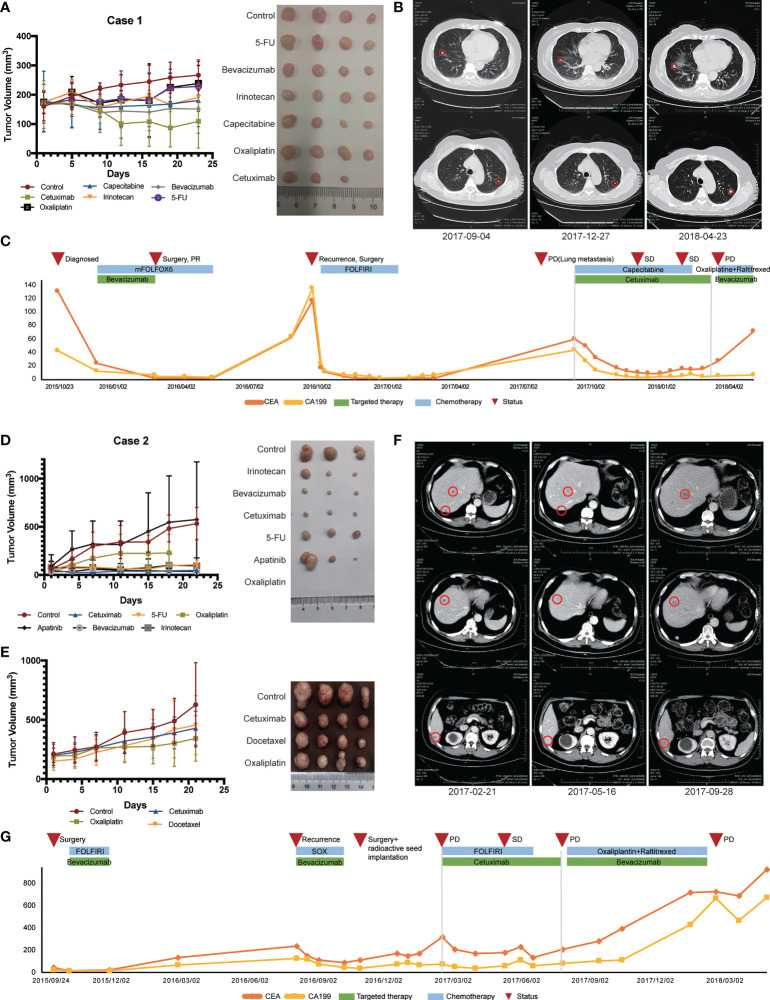
Therapeutic response of PDX models and corresponding patients. **(A)** *In vivo* drug sensitivity of Case 1 with KRAS mutation using PDX models. **(B)** Imaging changes of lung metastasis during treatment in Case 1. **(C)** Changes of serum tumor markers during treatment in Case 1. **(D)** The first *in vivo* drug sensitivity of Case 2 with KRAS mutation using PDX models. **(E)** *In vivo* drug sensitivity of Case 2 with KRAS mutation using PDX models after recurrence. **(F)** Imaging changes of liver metastasis during Ftreatment in Case 2. **(G)** Changes of serum tumor markers during treatment in Case 2.

Case 1 (No. CoZ0116) was first diagnosed with colon cancer in October 2015. After seven cycles of neoadjuvant therapy (mFOLFOX6 + bevacizumab), surgical treatment was performed. Postoperative pathology indicated PR, and chemotherapy with mFOLFOX6 regimen was continued postoperatively. However, in September 2016, the patient was diagnosed with a single liver metastasis and received the second surgery. After surgery, establishment of the PDX model was performed on liver metastatic specimens obtained by surgery. Drug sensitivity results showed that cetuximab, irinotecan, and 5-fluorouracil (5-FU) were sensitive (**Figure 3A**). According to the guideline of colorectal cancer, the patient was treated with FOLFIRI regime for 11 cycles due to the mutation of RAS gene. Regrettably, the patient presented with lung metastasis in September 2017. According to the PDX sensitivity results and the patient’s willingness to refuse intravenous chemotherapy, we selected cetuximab + capecitabine as an advanced line of treatment. Importantly, the patient’s lung metastases were controlled and a progression-free survival (PFS) of 6 months was achieved (**Figures 3B, C**).

Another patient (Case 2, CoY0011) was a 57-year-old man who first diagnosed with colon cancer in September 2015 and received surgical treatment. PDX model was successfully established after surgical treatment and tissue samples were obtained. Drug sensitivity test was initially conducted and showed that all groups, except for the apatinib group, inhibited tumor growth compared with control group that did not receive drug treatment (**Figure 3D**). According to the patient’s tumor stage and guidelines, the patient received FOLFIRI chemotherapy. No local recurrence or distant metastasis were found during the following 1 year’s periodic re-examinations. In September 2016, the patient was admitted to our hospital because of decreased appetite with fatigue and bone pain. Imageological examination and levels of serum tumor biomarkers both suggested the possibility of liver and bone metastases. In order to find out a better treatment plan, we resuscitated the PDX model and conducted drug sensitivity test again (**Figure 3E**). SOX (oxaliplatin + S-1) + bevacizumab was selected according to the results of two drug sensitivity tests. After four cycles of SOX + bevacizumab treatment, the levels of serum tumor biomarkers slightly decreased. In November 2016, the patient developed peritoneal metastasis, which revealed that our patient did not respond well to these therapeutic regimens. The antitumor effect of cetuximab was confirmed in both drug sensitivity tests, even though the patient had RAS mutation (**Figures 3D, E**). The patient was subsequently treated with cetuximab in combination with chemotherapy and achieved a PFS of 4 months (**Figure 3G**). **Figure 3F** shows the radiographic changes during cetuximab treatment.

## Discussion

The incidence of GI cancer is still increasing gradually, which is an important cause of cancer-related death (7). To date, several drugs have been presented for GI cancer with a certain efficacy. However, due to the strong individual heterogeneity, personalized precision treatment is still a favorable alternative for physicians and patients. The *in vitro* tumor model has been used as a standard tool for preclinical antitumor drug research, while the high failure rate of drugs has questioned the prediction ability of this traditional tumor model. Compared with the *in vitro* tumor model, the PDX model can better maintain the histopathological, genetic, and phenotypic characteristics of the tumor tissue, leading to enhance the prediction of the drug response (9, 17, 18). In recent years, a large number of PDX models have been established in various tumors, including gastric cancer (11, 16, 19), colorectal cancer (20), lung cancer (21), cervical cancer (22), etc. The PDX models have gradually become an effective tool for tumor biology research and anti-tumor drugs’ efficacy evaluation. However, the PDX models have not been widely used in clinical practice, mainly due to the instability of establishing PDX models.

Several factors including technicians’ skills contribute to the engraftment of PDX models. In our study, sample inoculation was initially carried out by five laboratory staff with at least 1 year of experience, minimizing the difference in engraftment rate. The successful establishment of PDX models can be influenced by experimental, clinicopathological, and molecular parameters. To date, few studies have reported the factors influencing engraftment rate of PDX models for GI cancer separately. Zhu et al. found no significant difference between transplantation rate and clinicopathological characteristics except for chemotherapy for gastric cancer (19). However, Zou et al. reported that transplantation rates of biopsied samples from stage III or IV (17.7%, 22/124) were significantly higher than those from early stage (0, 0/19, P < 0.05) in esophageal cancer (23). In colorectal cancer, a significantly higher successful PDX establishment rate was found in liver metastatic specimens than that in primary specimens (N=26; 76.7% *vs*. 57.7%). No clinicopathological features led to significant differences in the PDX establishment rate for metastatic colorectal cancer (20). However, no study has regarded GI tumors as a whole to study factors influencing PDX engraftment rate.

In our study, we identified a number of these factors associated with PDX engraftment. Colorectal cancer showed a higher engraftment rate (75.2%) compared with esophageal cancer (60.0%) or gastric cancer (39.6%). The results revealed that there were significant differences in the tumor engraftment rate of diverse digestive tract tumors. Among them, engraftment rate of stomach was the least, which could be related to the high heterogeneity of gastric cancer. Squamous cell carcinoma had a higher engraftment rate (63.6%), while SRCC, which is more common in gastric cancer, had a lower engraftment rate (25.9%). It is noteworthy that it is easier to establish a PDX model for moderately differentiated tumors than for poorly differentiated and well-differentiated tumors. It could be related to the fact that mesenchymal cells are less essential in moderately differentiated tumors than poorly differentiated tumors, while the tumor load in well-differentiated tumors might be extremely low to engraftment in PDX models. For instance, a previous study reported that poorly differentiated adenocarcinoma required the use of transforming growth factor-β (TGF-β) during stromal response, whereas human TGF-β may not interact with mouse stromal cells (24). In addition, we found that for patients who had received neoadjuvant therapy, the tumor engraftment rate of PD or SD specimens was significantly higher than that of PR specimens (65.4% *vs*. 29.4%, *P* = 0.021, **Figure 2**). This may explain the influence of the degree of malignancy of tumor on the tumor formation rate of PDX. In addition, PDX can be established for drug sensitivity test to screen effective drugs for patients who have failed in conventional neoadjuvant therapy. Another important finding is that although SRCC is considered a histological type with a highly malignant biological behavior (25–27), it has a significantly lower engraftment rate than other histological types in our study. The unique biological feature of SRCC is associated with the production and accumulation of abundant mucins in the cytoplasm and plasma membrane. Murakami H et al. found that their newly established SRCC cell lines grew retarded *in vivo* in nude mice and found inflammatory responses around subcutaneous tumors, possibly in response to extracellular mucin secretion. It suggested that this may not only be related to the low growth rate of the tumor cells, but also the inflammatory or immune responses of macrophages and natural killer cells to the host (28). We also considered that the low PDX engraftment rate of SRCC might be correlated with it.

The predictive value of data obtained from PDX-based studies in biomarker analysis is highly valuable for the PDX modeling in cancer research. Previous researches demonstrated that PDX models are biologically and genetically similar to primary tumors (29, 30). Our study mainly concentrated on some molecular characteristics related to prognosis and treatment, such as Ki67, HER-2, RAS, BRAF, and MMR status. Ki67 is widely recognized as a proliferation index, which is expressed in the cell nucleus during mitosis. Our data showed that a strongly positive Ki-67 could be correlated with a high engraftment rate. One possibility for the higher engraftment rate is that when tumor tissue samples are implanted into immunodeficient mice, cells with strongly positive Ki-67 own high proliferation capacity. Similar results were also reported in PDX models of other types of cancer (31–34). Besides, our results showed that a higher engraftment rate was observed in dMMR tumors, while no significant association was observed between other important gene mutations (e.g., KRAS and BRAF mutations) and engraftment rate.

Another approach to determine the value of PDX models in cancer research is analyzing the predictive value of the data obtained from PDX-based studies with consideration of drug efficacy and patient outcome. KRAS and BRAF are two downstream molecules of epidermal growth factor receptor (EGFR) and play important roles in EGFR signaling cascade. Activating mutations in KRAS exon 2 can induce infinite proliferation of tumor cells, thereby freeing the pathway from the control of EGFR (35, 36). The mutation status of RAS and BRAF genes is considered to be of great significance in guiding the treatment and predicting the prognosis of colorectal cancer patients (37–41). The development of cetuximab, a mouse/human chimeric monoclonal antibody against EGFR, bring new expectations to patients. The guidelines and studies generally recommended cetuximab therapy only to patients with RAS and BRAF wild-type colorectal cancer (42). In previous clinical studies, cetuximab had a significantly lower overall tumor response rate in patients with RAS mutation than in patients with RAS wild-type colorectal cancer. However, few patients with PR or CR were reported, which could be related to the heterogeneity of the tumor (43–46). In our study, several PDX models of KRAS mutant patients showed efficacy against cetuximab therapy. In the current study, we presented two typical patients with KRAS mutations who developed recurrence and distant metastasis after treatments. The drug sensitivity screening of PDX model showed that cetuximab had a certain efficacy, so that cetuximab was applied to the patients. The levels of tumor indicators (CA199 and CEA) significantly decreased in the two patients, and both patients achieved the best efficacy of PR with PFS reaching 6 and 4 months respectively. This suggests that cetuximab may have a promising effect on some patients with RAS or BRAF mutation, and some patients may lose the opportunity of undergoing effective targeted therapies because of the genetic test results. This further indicated that the establishment of a PDX model is of great importance for medication of patients, especially for the posterior line treatment of patients with recurrence and metastasis.

## Conclusions

In the present study, we successfully established a large-scale PDX model for GI tumors in our center. The relationship between clinicopathological and molecular features and engraftment rates were clarified. Furthermore, this resource provides us with profound insights into tumor heterogeneity, making these models valuable for PDX-guided treatment decisions, and offering the PDX model as a great tool for personalized treatment and translation research.

## Data availability statement

The raw data supporting the conclusions of this article will be made available by the authors, without undue reservation.

## Ethics statement

The studies involving human participants were reviewed and approved by the Ethics Committee of The First Affiliated Hospital, College of Medicine, Zhejiang University (No.2018-309 and IIT20221079A). The patients/participants provided their written informed consent to participate in this study. The animal study was reviewed and approved by the Animal Experimental Ethical Inspection of the First Affiliated Hospital, College of Medicine, Zhejiang University (No.2018-378). Written informed consent was obtained from the individual(s) for the publication of any potentially identifiable images or data included in this article.

## Author contributions

XY and YRC are responsible for the study concept and design. YRC and JL are responsible for the acquisition of data and the development of methodology. KH, YYC, KJ, and HYW are responsible for the tumor sample collection and establishment of PDX. XY, YRC, and YD are responsible for the analysis and interpretation of data. YRC, XY, and JL are responsible for the writing of the manuscript. HZ, HHW, and LT are responsible for the review and/or revision of the manuscript. All authors contributed to the article and approved the submitted version.

## Funding

This work was supported by grants from: Project of the regional diagnosis and treatment centre of the Health Planning Committee (No. JBZX-201903), Science and Technology Project of Zhejiang Province (2021C03119), Chinese Medicine Technology Project of Zhejiang Province (2020ZZ013) and Youth Project of Zhejiang Provincial Natural Science Foundation (LQ18H160012).

## Conflict of interest

The authors declare that the research was conducted in the absence of any commercial or financial relationships that could be construed as a potential conflict of interest.

## Publisher’s note

All claims expressed in this article are solely those of the authors and do not necessarily represent those of their affiliated organizations, or those of the publisher, the editors and the reviewers. Any product that may be evaluated in this article, or claim that may be made by its manufacturer, is not guaranteed or endorsed by the publisher.
